# Cyclin D1, Id1 and EMT in breast cancer

**DOI:** 10.1186/1471-2407-11-417

**Published:** 2011-09-28

**Authors:** Nicholas P Tobin, Andrew H Sims, Katja L Lundgren, Sophie Lehn, Göran Landberg

**Affiliations:** 1 Breakthrough Breast Cancer Research Unit, School of Cancer, Enabling Sciences and Technology, University of Manchester, Manchester Academic Health Science Centre, Paterson Institute for Cancer Research, The Christie NHS Foundation Trust, Wilmslow Road, Manchester, M20 4BX, UK; 2 Cancer Center Karolinska, Karolinska Institute and University Hospital, Stockholm, S-17176, Sweden; 3 Applied Bioinformatics of Cancer, Breakthrough Breast Cancer Research Unit, Edinburgh Cancer Research Centre, Institute of Genetics and Molecular Medicine, Crewe Road South Edinburgh, EH4 2XR, UK; 4 Department of Laboratory Medicine, Center for Molecular Pathology, Lund University, Malmö University Hospital, Malmö, SE-205 02, Sweden

**Keywords:** Cyclin D1, Id1, EMT, breast cancer, migration, recurrence-free survival, claudin-low

## Abstract

**Background:**

Cyclin D1 is a well-characterised cell cycle regulator with established oncogenic capabilities. Despite these properties, studies report contrasting links to tumour aggressiveness. It has previously been shown that silencing cyclin D1 increases the migratory capacity of MDA-MB-231 breast cancer cells with concomitant increase in 'inhibitor of differentiation 1' (*ID1*) gene expression. Id1 is known to be associated with more invasive features of cancer and with the epithelial-mesenchymal transition (EMT). Here, we sought to determine if the increase in cell motility following cyclin D1 silencing was mediated by Id1 and enhanced EMT-features. To further substantiate these findings we aimed to delineate the link between *CCND1*, *ID1 *and EMT, as well as clinical properties in primary breast cancer.

**Methods:**

Protein and gene expression of *ID1*, *CCND1 *and EMT markers were determined in MDA-MB-231 and ZR75 cells by western blot and qPCR. Cell migration and promoter occupancy were monitored by transwell and ChIP assays, respectively. Gene expression was analysed from publicly available datasets.

**Results:**

The increase in cell migration following cyclin D1 silencing in MDA-MB-231 cells was abolished by Id1 siRNA treatment and we observed cyclin D1 occupancy of the Id1 promoter region. Moreover, *ID1 *and *SNAI2 *gene expression was increased following cyclin D1 knock-down, an effect reversed with Id1 siRNA treatment. Similar migratory and *SNAI2 *increases were noted for the ER-positive ZR75-1 cell line, but in an Id1-independent manner. In a meta-analysis of 1107 breast cancer samples, *CCND1*^low^/*ID1*^high ^tumours displayed increased expression of EMT markers and were associated with reduced recurrence free survival. Finally, a greater percentage of *CCND1*^low^/*ID1*^high ^tumours were found in the EMT-like 'claudin-low' subtype of breast cancer than in other subtypes.

**Conclusions:**

These results indicate that increased migration of MDA-MB-231 cells following cyclin D1 silencing can be mediated by Id1 and is linked to an increase in EMT markers. Moreover, we have confirmed a relationship between cyclin D1, Id1 and EMT in primary breast cancer, supporting our *in vitro *findings that low cyclin D1 expression can be linked to aggressive features in subgroups of breast cancer.

## Background

Cyclin D1 along with its binding partners CDK 4/6 partially mediate G1 to S-phase transition of the cell cycle through phosphorylation and inactivation of retinoblastoma (Rb) protein with subsequent release of E2F transcription factors [1-3]. The oncogenic activities of the protein have been addressed in numerous studies, [4-7] and many human cancers including breast, colon, and prostate, overexpress cyclin D1 [8-10]. More recently, a number of cyclin D1 studies in breast cancer have focused on functions that are not directly related to cell cycle maintenance. Cyclin D1 can modulate the activity of transcription factors and histone deacetylase [11], it can activate oestrogen receptor in the absence of oestrogen [12], and it can bind to the upstream regulatory region of the diverse *Notch1 *gene [13]. Previous work by our group revealed a novel induction of breast cancer cell migration after cyclin D1 silencing, which may account for a worse clinical outcome for patients with low expression of the protein [14]. Of the genes upregulated following this silencing, Inhibitor of differentiation 1 (Id1), a basic helix-loop helix (bHLH) family member, represents a potential candidate modulating the effect of cyclin D1 on cell migration.

The four Id proteins (termed 1-4) represent the class V subgroup of the bHLH family, however in contrast to other bHLH transcription factors (that modulate gene expression though dimerization and DNA binding of canonical E-box promoter regions in target genes [15]), the Id proteins lack a DNA binding domain and instead bind to other bHLH family monomers, negatively regulating their activity [16]. Id1 has been associated with breast cancer progression in a number of studies. *ID1 *promoter regulation is lost in aggressive breast cancer cells [17], Id1 is associated with induction of cell proliferation and invasion [18], and stable antisense targeting of Id1 represses an aggressive and metastatic phenotype in mammary epithelial cells [19]. Recent data has also revealed that cyclin D1 binds to the *ID1 *promoter region in the mammary gland, and negatively regulates its transcription in mouse retina [13]. Given the role of Id1 in cell invasion and metastasis, it represents a strong candidate for driving breast cancer cell migration following cyclin D1 silencing.

Increased motility and invasiveness are inherent properties of a mesenchymal phenotype [20], and the process whereby a non-motile epithelial cell procures these traits is termed epithelial to mesenchymal transition (EMT). Recently, a role for EMT in the process of cancer metastasis has been postulated, and direct evidence of EMT has been demonstrated in a mouse mammary tumour model [21]. A number of distinct changes occur during the transition to a mesenchymal phenotype, most notably the down-regulation of epithelial markers such as E-cadherin, and an upregulation of mesenchymal markers including Snail, Slug, vimentin, Twist and fibronectin [22]. In addition, a number of phenotypic changes occur including loss of cell polarity and tight junction regulation, accompanied by cytoskeletal changes [23,24] and enhanced cell migration/invasion [25]. Id1 has previously been implicated with EMT both directly, through suppression of E-cadherin and zonula occludins-1 (ZO-1), in human kidney cells [26] and indirectly, through loss of Krueppel-like factor 17 (KLF17) in breast cancer cells [27]. As such, we wished to clarify whether the increase in cell migration following cyclin D1 silencing was due to an Id1-dependent increase in EMT markers.

In this study, we demonstrate that silencing Id1 prevents the cyclin D1 mediated increase in MDA-MB-231 breast cancer cell migration. We have identified that an increase in *SNAI2 *mRNA expression following cyclin D1 silencing is abolished in cyclin D1/Id1 double knock-down cells. A meta-analysis of primary breast tumours revealed significant associations between *CCND1*, *ID1*, *CDH1 *(E-cadherin) and recurrence-free survival. *CCND1 *and *ID1 *gene expression was also correlated with EMT-associated genes including, *VIM*, *SNAI1*, *SNAI2*, and *TWIST1*. Finally, the recently established claudin-low subtype of breast cancer, which is enriched in EMT markers, was found to have a four-fold greater proportion of *CCND1*^low^/*ID1*^high ^tumours compared to other breast cancer subtypes.

## Methods

### Cell culture

The human breast cancer cell lines MDA-MB-231 and ZR75-1 (ATCC, Int., Manassas, VA) were maintained in RPMI 1640 medium supplemented with 10% fetal calf serum (FCS), sodium pyruvate (1 mM) and 1 × PEST (streptomycin 90 μg/ml, penicillin 90 IU/ml). Cells were maintained in a humidified atmosphere of 5% CO_2_/95% air at 37°C.

### siRNA and vector transfections

7.5 × 10^5 ^cells were seeded in a 10 cm (47.16 cm^2^) culture dish with PEST-free serum-containing media (SM) for 24 h. The media was subsequently removed and PEST-free serum-free media (SFM) added along with 1 ml siRNA solution (OptiMEM, Gibco, Lipofectamine 2000, Invitrogen Life Technologies, Carlsbad, CA) giving a final concentration of 40 nM oligonucleotides. 5 h after transfection, SFM was replaced with SM and cells were allowed to grow for 20 h before harvesting for migration assay or western blot. ON-TARGET*plus *SMARTpool siRNA targeting cyclin D1, Id1 or Slug (Dharmacon RNA Technologies, Lafayette, CO) were included as standard experimental protocol. A non-targeting pool was used as negative control. For vector experiments, cells were treated as above with the following exceptions: seeding density was 11 × 10^5 ^cells in a 47.16 cm^2 ^culture dish and 1.5 μg of Id1 vector pCMV-SPORT6 or control vector pCMV6 was used (Invitrogen Life Technologies, Carlsbad, CA).

### Western blotting

Western blot was performed as previously described [28] with the following antibodies: anti-cyclin D1 (1:500, DCS-6, DAKO, Denmark), anti- Id1 (1:400, C-20, Santa Cruz), and anti-Actin (1:1000, I-19, Santa Cruz, CA, USA) Proteins were visualized with horseradish peroxidase conjugated secondary antibodies using the enhanced chemiluminescence detection system (Amersham Pharmacia Biotech, Little Chalfont, UK).

### Migration assays

Cell migration was routinely carried out in 8 μm-pore polycarbonate membrane Transwell chambers with a diameter of 6.5 mm (Corning, Inc. Corning, NY). The membranes were incubated in 150 μl serum-free RPMI 1640 for an initial equilibrium period of 1 h. Cells were resuspended in serum-free medium (1 × 10^6 ^cells/ml) and 100,000 cells were added to each migration chamber. The chambers were placed into wells containing 600 μl 10% FCS medium and cells were allowed to migrate for 4 h after siRNA or vector transfections. Cells remaining in the chamber were removed with a cotton swab and the migrated cells situated on the lower side of membranes were fixed for 15 min in PBS containing 4% paraformaldehyde. Membranes were cut and mounted on glass slides for DAPI staining and counted using a fluorescent microscope (cells in three 10X-magnification fields representing the composition of the membrane were counted). Assays were performed in triplicate with two migration membranes for every treatment, and the total number of migrated cells was used for graphical and statistical purposes.

### qPCR and Chromatin immunoprecipitation assay (ChIP)

Total RNA was isolated using an RNeasy Plus Kit (Qiagen, West Sussex, UK). RNA was eluted and quantified using a Nanodrop spectrometer (ThermoScientific, Leicestershire, UK). The reverse transcription step was performed using the TaqMan Reverse Transcription Reagent Kit (Applied Biosystems, CA, USA) according to manufacturer's guidelines. TaqMan real time PCR was designed using the Universal Probe Library (Roche Diagnostics, West Sussex, UK). Primers and sequences can be found in Additional File 1. RT-PCR was performed with 5 ng template cDNA using Taqman Master Mix (Applied Biosystems, CA, USA) and an ABI prism 7900 HT sequence detection system (Applied Biosystems, CA, USA). ChIP assay was carried out using the MAGnify™ Chromatin Immunoprecipitation System (Invitrogen Life Technologies, Carlsbad, CA). 3 μg of anti-cyclin d1 antibody (DCS-6, DAKO, Denmark) was used to pull down cyclin D1, with subsequent detection of Id1 using SimpleChIP™ Human Id1 Promoter Primers (Cell Signalling Technology, Danvers, MA) and Mrg1 as positive control (Eurofins Laboratories Ltd., Manchester, UK). Promoter occupancy was calculated based on the ratio of ChIP to input control.

### Microarray analysis

Gene expression analysis of cyclin D1 silenced cells was described previously [14]. All data is MIAME compliant and raw data has been deposited at the NCBI Geo database (Accession number GSE27260). A meta-analysis of six Affymetrix gene expression datasets comprising a total of 1107 primary human breast cancers was performed as previously described [29]. Clinicopathological features were retrieved from the original studies. The follow up endpoints for the Chin *et al.*, Pawitan *et al. *and Sotoriou *et al. *datasets were recurrence-free survival, whereas for Desmedt *et al.*, Ivshina *et al. *and Wang *et al. *datasets it was disease-free survival. The gene expression datasets of Herschkowitz *et al. *[30] and Neve *et al. *[31] were used to compare expression of *ID1*, *CCND1 *and EMT-related genes across the breast cancer subtypes, including claudin-low as determined by the study and breast cell lines respectively.

### Statistical methods

To examine the statistical significance of the differences seen in the Boyden migration and qPCR a two-tailed student's t-test was employed, assuming unequal variance. Unless noted otherwise, the standard error of the mean is stated. Statistical analyses were performed using SPSS software (version 17.0, SPSS, Chicago, IL, USA). For examination of the statistical significance of associations between *CCND1*, *ID1 *and other categorical variables, Spearman's rank-order correlation coefficient, Kruskal-Wallis and Wilcoxon/Mann-Whitney tests were used as indicated in figure legends. To study recurrence-free survival the Kaplan-Meier method was employed and to compare recurrence-free survival among different quartiles the log-rank test was used. For claudin-low subtype comparison a Chi^2 ^test was employed.

## Results

### Cyclin D1 and Id1 in breast cancer cell migration

**Figure 1:**
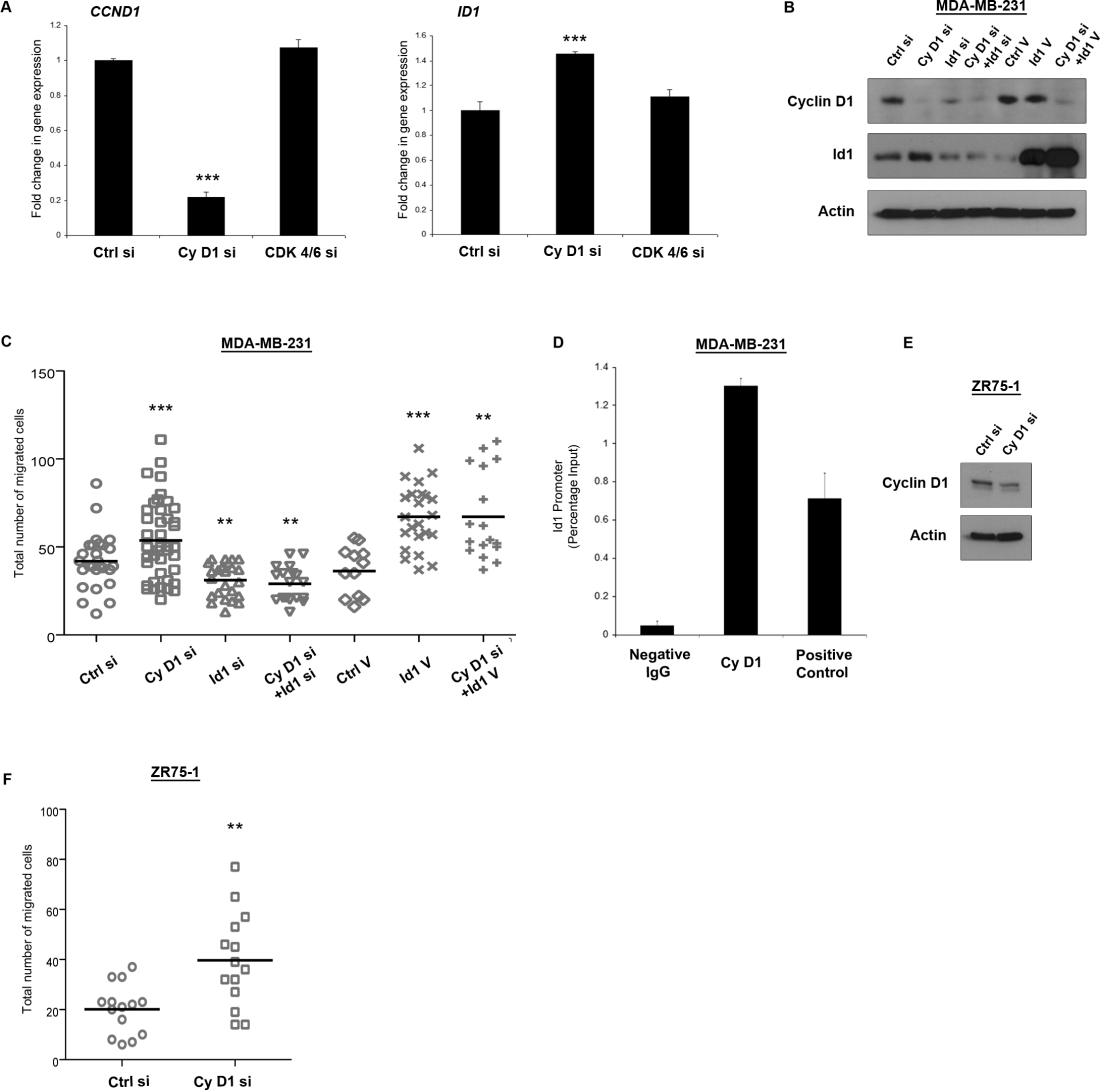
**Effect of cyclin D1 and Id1 on breast cancer cell protein expression and migration**. Actively cycling MDA-MB-231 and ZR75-1 cells were monitored 20 h post-transfection with the indicated siRNA (cyclin D1/CDK4/6/Id1) or vector (Id1) for changes in gene or protein expression, and migration. Blots are representative, and plots are mean values from at least three independent experiments. Error bars represent standard deviation. MDA-MB-231 cells: **(A) **Microarray analysis. Left panel: *CCND1 *gene expression, right panel: *ID1 *gene expression **(B) **Western blot for cyclin d1, Id1 and Actin protein, **(C) **Cell migration as measured by Boyden chamber assay, dots indicate total number of migrated cells. **(D) **ChIP assay for Id1 promoter region following cyclin D1 pull down. ZR75-1: **(E) **Western blot for cyclin d1 and Actin protein **(F) **Cell migration- Boyden chamber assay. ***P ≥ 0.001, **P ≥ 0.01, *P ≥ 0.05 vs. control, two-tailed student's t-test.

We have previously shown that cyclin D1 silencing increases migration of the ER-negative MDA-MB-231 breast cancer cell line, an effect not observed when silencing its binding partners CDK 4/6 (Lehn et al. 2010). *ID1 *was among the genes most significantly upregulated (Figure 1A, 1.40 ± 0.007 fold) in response to cyclin D1 knock-down (Figure 1A, 0.22 ± 0.017 fold) and was unchanged following CDK 4/6 silencing. As a role for Id1 in breast cancer cell metastasis and aggressiveness has previously been suggested [17], it was logical to examine whether it was also responsible for the cyclin D1 induced increase in cell migration. Western blotting confirmed an increase in Id1 protein following cyclin D1 siRNA treatment, and effective cyclin D1 and Id1 silencing (Figure 1B). In addition, neither Id1 silencing nor vector overexpression altered cyclin D1 protein levels after 24 h.

Boyden chamber migration assays accurately replicated previous experimental results, with cyclin D1 siRNA treatment of MDA-MB-231 cells increasing the total number of migrated cells to 53.57 ± 3.5, compared to control levels of 41.89 ± 3.0 (Figure 1C). Notably, Id1 siRNA decreased cell migration (31.13 ± 1.99), and addition of cyclin D1 siRNA was unable to significantly rescue this effect (46.00 ± 2.34). Overexpression of Id1 increased migration (67.13 ± 3.82 compared to control of 36.33 ± 4.00) and similar effects were found when treating cells with both Id1 vector and cyclin D1 siRNA (67.24 ± 6.08). To discount the possibility that increased siRNA concentration may have a negative impact on migration in the cyclin D1 and Id1 siRNA treated cells, we assessed single and double concentrations of siRNA in control cells and found no significant difference in cell migration (data not shown). To determine if Id1 could be a transcriptional target of cyclin D1 in MDA-MB-231 cells, we performed a ChIP assay, and demonstrated that cyclin D1 occupancy in the Id1 promoter was significantly higher (1.30% of input) in cyclin D1 pull-down than in a negative mouse IgG control, and higher still than the positive control Mrg1 (Bienvenu et al. 2010) (Figure 1D).

We next examined whether cyclin D1 silencing could effect migration in an ER-positive breast cancer cell line with similar cyclin D1 levels to MDA-MB-231 cells. siRNA treatment against cyclin D1 reduced its protein levels (Figure 1E) and also significantly increased migration (39.71 ± 5.04 compared to control of 20.14 ± 2.66, Figure 1F) of ZR75-1 cells. However, given the extremely low protein expression levels of Id1 in ZR75-1 cells, it is unlikely that the increase in migration is mediated through Id1 in this cell line. In addition to the interaction we have demonstrated between cyclin D1 and Id1, other regulators of Id1 have been previously identified. TGF-beta [32], KLF17 [33] and Src [34] are all known to interact and influence Id1 expression. Thus, levels of Id1 protein in ZR75-1 cells may reflect interactions with other transcriptional regulators. To directly address this, we examined TGF-beta (a known inducer of Id1 [32]) gene expression in a range of breast cancer cell lines and noted high levels in MDA-MB-231 cells relative to ZR75-1 cells (Additional file 2). Importantly, none of the aforementioned transcripts were altered in our expression array data in response to cyclin D1 silencing and are hence unlikely to contribute to the migratory effect we have observed.

Together, these data indicate that the increase in Id1 following cyclin D1 silencing in MDA-MB-231 cells is responsible for their enhanced migratory capacity, but that this does not appear to be the only mechanism by which cyclin D1 can induce cell migration. Mounting evidence has indicated the occurrence of an EMT-like phenotype in migratory breast cancer cells [35,36]. Given this evidence we wished to determine whether the Id1 induced increase in migration following cyclin D1 silencing may be mediated through enhanced features of EMT.

### Cyclin D1 silencing in MDA-MB-231 cells increases EMT gene expression in an Id1 dependent manner

**Figure 2:**
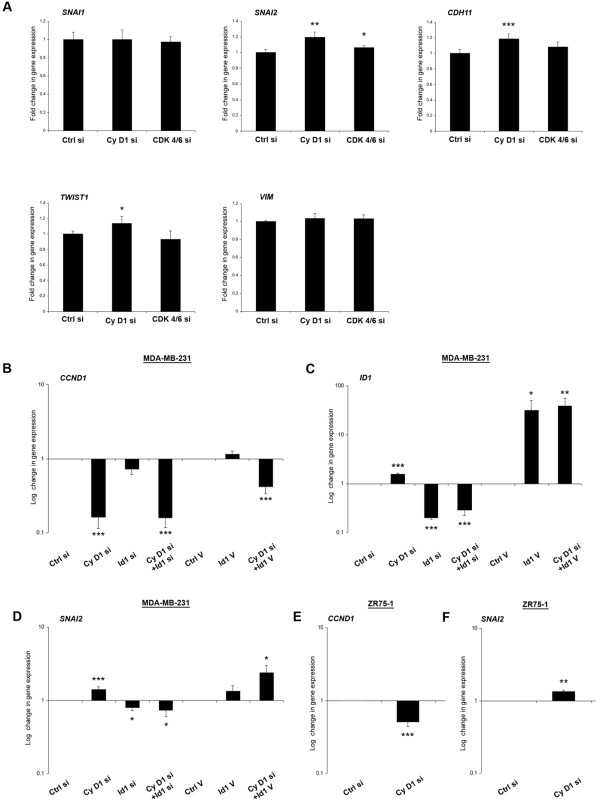
**Effect of cyclin d1 and Id1 on EMT markers**. MDA-MB-231 cells were monitored 20 h post-transfection with the indicated siRNA (cyclin D1/CDK4/6/Id1) or vector (Id1) for changes in EMT-related gene expression by microarray analysis. Additionally, MDA-MB-231 and ZR75-1 gene expression was examined by qPCR assay. Plots are mean values from at least three independent experiments Error bars represent standard deviation. **(A) **Microarray analysis of *SNAI1*, *SNAI2*, *CDH11*, *TWIST1 *and *VIM *gene expression. **(B-D) ***CCND1, ID1 *and *SNAI2 *in MDA-MB-231 cells. **(E, F) **qPCR analysis of *CCND1 *and *SNAI2 *in ZR75-1 cells. ***P ≥ 0.001, **P ≥ 0.01, *P ≥ 0.05 vs. control, two-tailed student's t-test.

Examination of EMT-related genes in the microarray analysis of MDA-MB-231 cells showed significant increases in *SNAI2 *(1.19 ± 0.03 fold), *CDH11 *(OB-Cadherin, 1.18 ± 0.03 fold), and *TWIST1 *(1.13 ± 0.05 fold), following cyclin D1 silencing. A modest increase in *SNAI2 *(1.06 ± 0.01 fold) expression was noted after CDK4/6 silencing, but neither siRNA treatment had an effect on *SNAI1 *or *VIM *expression (Figure 2A).

Using siRNA against cyclin D1 and Id1 we confirmed significantly decreased levels of *CCND1 *by qPCR, and found that Id1 siRNA had no significant impact on *CCND1 *expression (Figure 2B) after 24 h. Increased *ID1 *levels (1.58 ± 0.09 fold) were noted following cyclin D1 silencing (Figure 2C) and the effect of Id1 siRNA on *ID1 *expression was reduced when combined with cyclin D1 siRNA (0.18 ± 0.01 vs. 0.28 ± 0.06 fold respectively, *P = *0.019). As noted in our microarray data, cyclin D1 silencing increased *SNAI2 *levels, a result validated by qPCR analysis (1.41 ± 0.1 fold). This increase was reversed when cyclin D1 was silenced in combination with Id1 (0.73 ± 0.13 fold of control, Figure 2D). Id1 overexpression increased *SNAI2 *levels (1.34 ± 0.22 fold), an effect greatly enhanced when cyclin D1 was also silenced (2.39 ± 0.64 fold). Notably, silencing of cyclin D1 was unable to increase MDA-MB-231 cell migration when Slug was also silenced (Additional File 3). We also observed an increase in *SNAI2 *expression following cyclin D1 silencing (Figure 2E) in ZR75-1 cells (1.34 ± 0.05 fold, Figure 2F).

These results suggest a novel effect whereby cyclin D1 silencing enhances a mesenchymal phenotype in MDA-MB-231 and ZR75-1 cells. In order to further validate our hypothesis, we next examined gene expression data from a large cohort of breast cancer patients.

### *CCND1 *and *ID1 *expression are correlated to clinicopathological parameters and predict recurrence risk in breast cancer

To investigate the relationship between *CCND1 *and *ID1 *expression in primary breast tumours we used a previously published meta-analysis consisting of six groups of tumours on Affymetrix arrays totaling 1 107 samples. Due to the large number of patients and spread of gene expression values we quartiled each gene, giving us the following subgroups- 1 (low expression), 2 (low-medium), 3 (medium-high) and 4 (high). Initial examination of clinicopathological parameters revealed that *ID1 *was negatively correlated to tumour grade (p < 0.001), and size (p = 0.005). *CCND1 *expression was associated with ER-positive breast cancers (p < 0.001), and lower histological grade (p = 0.002) (Table 1). Neither *CCND1 *nor *ID1 *provided independent prognostic information in a Cox multivariate analysis (data not shown).

**Table 1:** Distribution of *CCND1 *and *ID1 *gene expression according to clinico- pathological parameters in breast cancer patients

	*CCND1*					*ID1*				
**Variable**	**1** **N = 277**	**2** **N = 276**	**3** **N = 277**	**4** **N = 277**		**1** **N = 277**	**2** **N = 276**	**3** **N = 277**	**4** **N = 277**	

**ERα positive (%)** *P*- value (R-value)					< 0.001† (0.306)					0.529† (0.021)
< 10	118	49	44	28		61	66	52	60	
≥ 10	117	177	195	211		172	170	182	176	
Missing cases: 168										

**NHG** *P*- value (R-value)					0.002 (-0.098)					< 0.001 (-0.199)
I	27	48	60	32		22	41	44	60	
II	72	79	86	93		77	78	87	88	
III	96	73	53	65		90	84	62	51	
Missing cases: 323										

**Lymph node status** *P*- value (R-value)					0.360† (0.030)					0.078† (-0.058)
N = 0	192	198	200	190		190	190	197	203	
N > 0	41	30	39	47		43	48	33	33	
Missing cases: 170										

**Tumour size, mm** *P*- value (R-value)					0.415† (-0.032)					0.005† (-0.109)
≤ 20	71	90	97	80		63	91	89	95	
> 20	90	75	73	86		95	83	69	77	
Missing cases: 445										

* Correlations were calculated using Spearman's ρ unless otherwise specified

† Wilcoxon/Mann-Whitney test (two-sided)

ER, Oestrogen receptor; NHG, Nottingham Histologic Grade.

**Figure 3:**
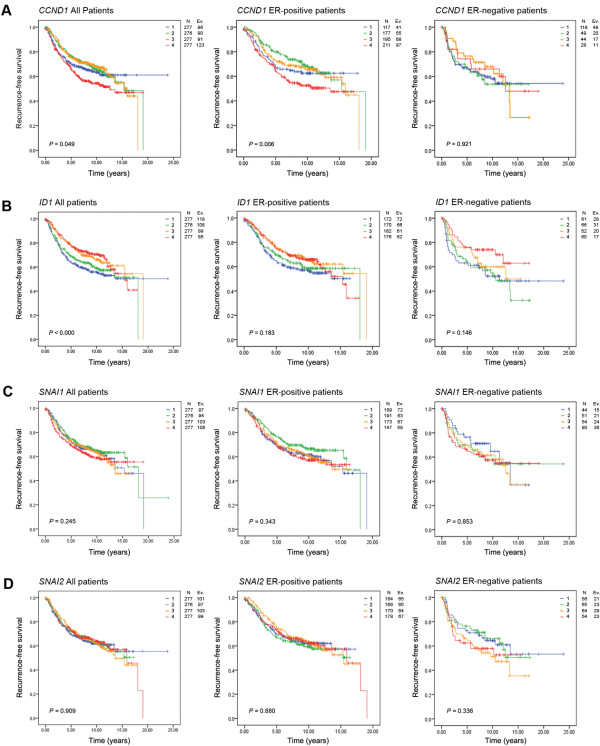
**Correlation of *CCND1*, *ID1*, *SNAI1 *and *SNAI2 *expression to recurrence free survival**. Expression of our genes of interest in relation to recurrence-free survival was examined in a breast cancer database containing 1,107 tumours from Sims *et al. *(2008). Gene expression intensity was quartiled as 1-low, 2- medium low, 3- medium high and 4- high, and assessed in all patients, ER-positive and ER-negative patients, respectively **(A) ***CCND1 *quartiles **(B) ***ID1 *quartiles **(C) ***SNAI1 *quartiles **(D) ***SNAI2 *quartiles. P-value is based on log-rank test.

Next, we determined how these quartiles related to recurrence-free survival (RFS) in the combined datasets. In all patients, and particularly in the subgroup of ER-positive patients, high expression of *CCND1 *was associated with the shortest RFS (p = 0.049 and p = 0.006, respectively, Figure 3A, left and middle panels, log-rank test). This effect was not observed in the ER-negative subgroup (Figure 3A, right panel). Conversely, low *ID1 *expression was associated with the shortest RFS in all patients (p < 0.001, Figure 3B, left panel), but not in the ER-positive and negative subgroups (Figure 3B, middle and right panels). The levels of EMT-related genes, *SNAI1 *(Figure 3C), *SNAI2 *(Figure 3D), *VIM *or *TWIST *(Additional File 4B and 4C) were not of significant prognostic value. However, *CDH1 *(E-cadherin) significantly predicted RFS in all and ER-positive patients (Additional File 4A, left and middle panels).

### Low *CCND1 *and high *ID1 *expressing tumours show increased EMT-related gene expression and predict risk of recurrence in breast tumours

**Figure 4:**
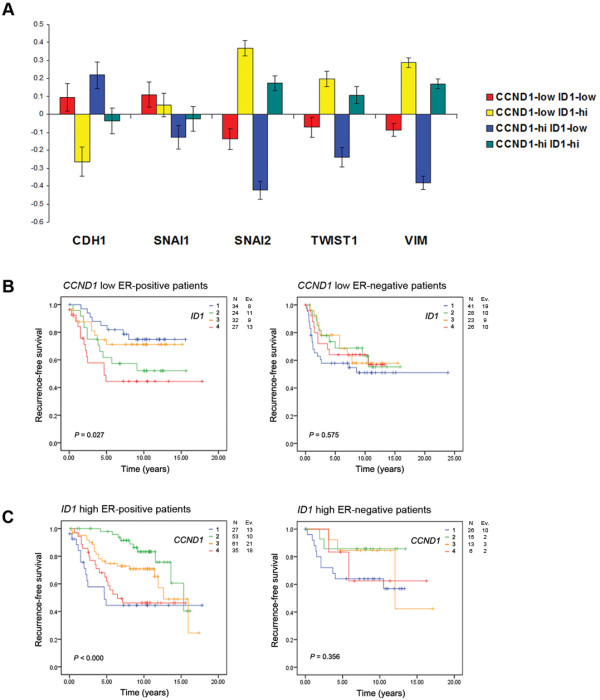
**EMT-related gene expression intensity and recurrence free survival in *CCND1*/*ID1 *high and low tumours**. A breast cancer database was employed to examine **(A) **Mean-centered average expression of EMT-related genes of interest in *CCND1*/*ID1 *subgroups, and **(B, C) **Recurrence-free survival of ER-positive and negative patients in *CCND1*/*ID1 *subgroups. P-value is based on log-rank test.

As our *in vitro *experiments indicated that *CCND1^low^/ID1^high ^*breast cancer cells exhibit increased invasion and expression of the *SNAI2 *gene, and our survival analysis indicated that low *CCND1 *and high *ID1 *expression can predict RFS in breast cancer patients; we examined all four combinations of *CCND1*^low/high ^and *ID1 *^low/high ^gene expression in relation to well-characterised EMT genes in all patients of the same tumour material. The highest expression of *SNAI2*, *TWIST1*, *VIM *and lowest expression of *CDH1 *was found in the *CCND1^low^*/*ID1^high ^*subgroup of tumours (Figure 4A, yellow bars). Further weight was added to this analysis when examining the *CCND1*^low/high^/*ID1*^low ^subgroups of tumours (Figure 4A, red and blue bars, respectively). These tumours encompass the lowest expression of *SNAI2*, *TWIST1*, *VIM *and highest expression of *CDH1*. This suggests, as our MDA-MB-231 *in vitro *experiments demonstrated, that cyclin D1 is unable to influence the induction of EMT in the absence of Id1.

To gain further insight into the relationship between cyclin D1 and Id1 we examined the *CCND1*^low^/*ID1*^high ^subgroups with regards to RFS in all, ER-positive, and negative patients. No statistical significance was found when examining all (data not shown) or ER-negative patients however, high *ID1 *expression was associated with the shortest RFS (Figure 4B, left and right panels) in *CCND1*^low ^ER-positive tumours. In addition, both low and high *CCND1 *expression was associated with the shortest RFS in *ID1*^high ^ER-positive tumours with no statistical significance observed in all (data not shown) or ER-negative patients (Figure 4C, left and right panels).

### Low *CCND1 *and high *ID1 *expression is dominant in the EMT-related basal B breast cancer cell lines and claudin-low subtype of tumours

**Figure 5:**
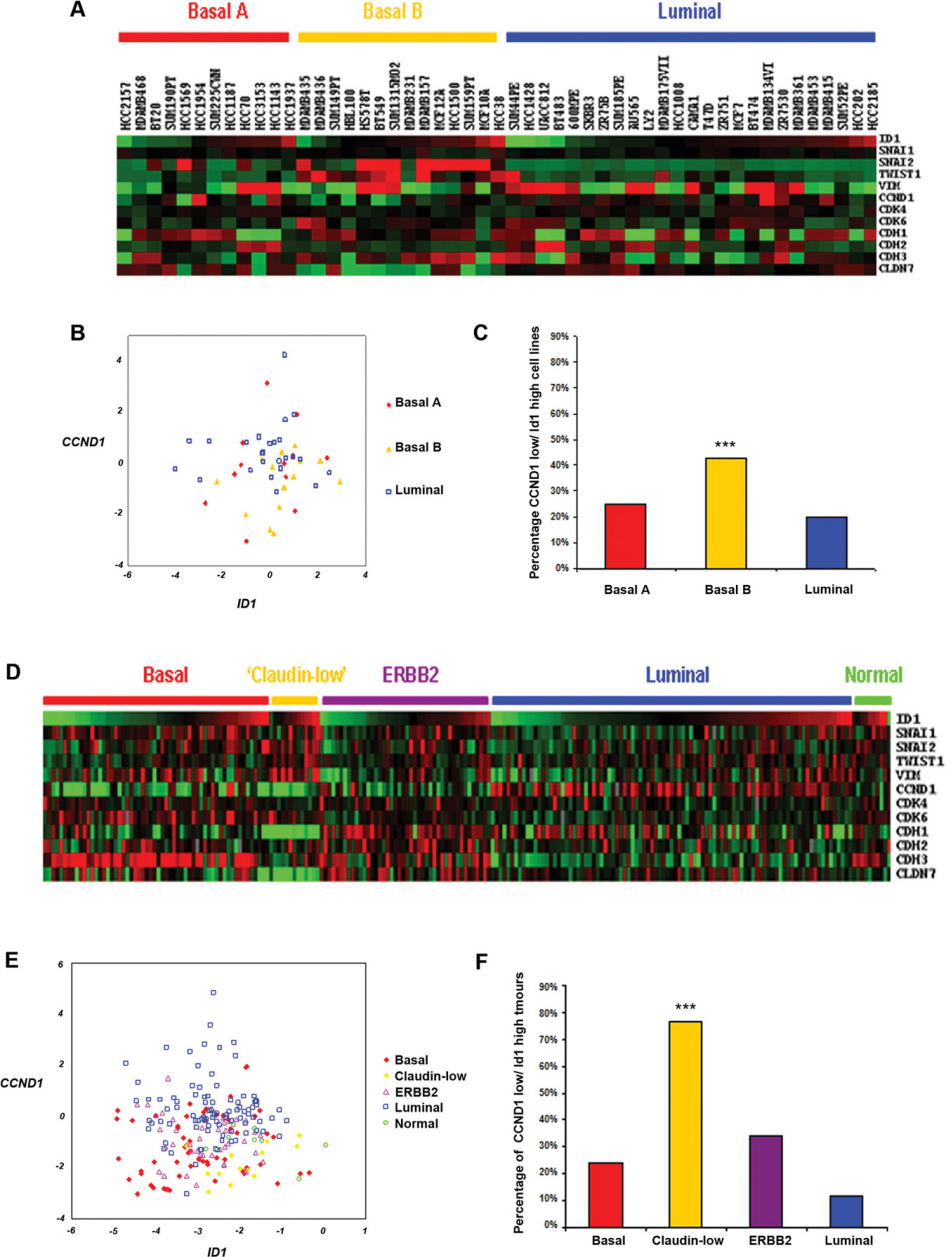
**Gene expression of Cyclin d1/Id1 and EMT markers in breast cancer cell lines and claudin-low tumours**. Expression of EMT and related genes from the Neve *et al *cell line **(A-C) **and Herschkowitz *et al. *claudin-low **(D-F) **studies. **(A and D) **Heatmaps showing relative expression of genes of interest, Red = high, Green = low. **(B and E) **Scatterplots showing the relationship between breast cancer subgroups and *CCND1*/*ID1 *expression **(C and F) **Proportion of *CCND1-*low/*ID1*-high cell lines and tumours in breast cancer subgroups. ***P ≥ 0.001, Chi^2 ^test.

A number of studies [31,37-39] have consistently split breast cancer cell lines into three groups based on their gene expression profiles; luminal, basal (or basal A) and mesenchymal/basal B/claudin-low subtypes. A greater proportion of the mesenchymal/basal B/claudin-low cell lines have low *CCND1 *and high *ID1 *expression than luminal (including ZR75-1) or basal A subtypes (Figure 5A-C) in the Neve *et al. *dataset (profiles for the same cell lines are highly correlated across several independent studies, data not shown) and have increased expression of EMT markers (*SNAI1*, *SNAI2*, *TWIST1*, *VIM*), along with low *CDH1*. Although ZR75-1 cells have similar levels of *CCND1*, they are of luminal subtype and display high expression of *CDH1 *(Figure 5A). This demonstrates the distinct difference between these cells lines, which may explain why Id1 appears unnecessary for enhanced EMT features in ZR75-1 cells following cyclin D1 silencing.

Recent studies have identified a 'claudin-low' subtype of breast cancer amongst human tumours through gene transcriptional profiling [30,40,41]. Tumours of the claudin-low subtype putatively contain tumour initiating cells (TIC), display high expression of EMT markers, and are believed to be the closest representation of an EMT phenotype in breast cancer [42]. This subtype displayed the highest proportion of *CCND1^low^/ID1^high ^*expressing tumours (Figure 5D-F). These data are consistent with our observation that *CCND1*^low^/*ID1*^high ^tumours belong to a subgroup of breast tumours with distinct expression pattern of *CCND1*, *ID1 *and EMT related genes.

## Discussion

In this study we demonstrate that the increase in MDA-MB-231 cell migration following cyclin D1 silencing is dependent on the upregulation of Id1. Previous studies have found both similarities and differences to our experimental model. Caldon *et al. *showed an increase in Id1 protein in mouse mammary epithelial cells isolated from cyclin D1^-/- ^mice compared to wild type, in line with our observations. Moreover, they also established the inability of Id1 to promote proliferation of mammary acini in the absence of cyclin D1 [43]. Swarbrick *et al. *revealed a decrease in cyclin D1 expression 48 h after Id1 silencing in MCF7 cells [44], and others report the same effect in both MCF7 and MDA-MB-231 cells [45]. We did not observe this decrease in cyclin D1 protein expression in MDA-MB-231 cells after 24 h in our study. However, qPCR analysis showed a similar decrease in cyclin D1 mRNA levels which may become more apparent on the protein level after 48 h. Bienvenu *et al. *demonstrated binding of cyclin D1 to the promoter region of *ID1 *in mouse retinal cells, and when comparing wildtype to *CCND1*^-/- ^mice found an 8-fold enrichment of *ID1*. We have also observed occupancy of the Id1 promoter region by cyclin D1 in MDA-MB-231 cells, where it may repress Id1 expression. These data demonstrate the complex relationship between cyclin D1 and Id1. It is important to note that here we are only proposing this mechanism in MDA-MB-231 cells and in a distinct subset of representative breast tumours. We observed this complexity during the course of our work, where despite an increase in ZR75-1 migration following cyclin D1 silencing, Id1 protein levels were so low as to not substantially contribute to this effect. We postulate that in ZR75-1 cells other known transcription regulators of Id1 such as TGF-beta may be responsible for repressing expression of the protein. Importantly, TGF-beta and other known Id1 regulators (KLF17, Src) were unchanged in our MDA-MB-231 microarray following cyclin D1 silencing, indicating they do not contribute to the upregulation of Id1 or migration in our analysis.

It is pertinent to highlight that the increase in migration we have observed is occurring in an already highly invasive, mesenchymal-like cell line. This may account for a lessened migratory response to cyclin D1 silencing. Further evidence of this concept is shown in the more epithelial-like, less typically invasive ZR75-1 cells, where the increase in cell migration is more pronounced (1.89 fold vs. 1.3 fold in MDA-MB-231) following cyclin D1 knock-down. In addition, cyclin D1 is known to be expressed at variable levels across cell lines and subtypes of breast cancer thus, silencing of cyclin D1 is unlikely to increase migration uniformly in all cell types.

A common feature in our MDA-MB-231 and ZR75-1 cells was an increase in *SNAI2 *expression 24 h after cyclin D1 knock-down, which coincided with an increase in cell migration. In MDA-MB-231 cells, silencing of Id1 reversed this and *SNAI2 *expression was decreased, as was cell migration. Moreover, silencing of Slug- the *SNAI2 *protein, significantly decreased MDA-MB-231 migration, and cyclin D1 silencing was unable to rescue this effect. These migratory observations for *SNAI2 *are in line with previous experimental data, indicating that Slug expression induces a migratory phenotype and can represses E-cadherin, inducing an EMT in epithelial cells [46]. Moreover, siRNA against Slug decreases MDA-MB-231 cell migration [47], and Slug and Snail are overexpressed invasive ductal carcinoma [48]- a form of breast cancer hallmarked by cell migration. In our experimental model, Slug would appear a likely candidate mediating the observed migratory effects, however it is entirely plausible that it does so in conjunction with other EMT factors. We also found statistically significant changes in *TWIST1 *and *CDH11 *(the positive EMT-regulator also known as OB-cadherin) following cyclin D1 silencing, both of which have been implicated with enhanced cell motility [49,50]. The changes in our EMT markers are in the order of 1.13 to 1.19 fold of control by expression array analysis (Figure 2A). We note that these figures are more meaningful when taken in the context of the most increased gene in our expression array, which was only upregulated 1.8 fold [14]. As may be expected from treatment with siRNA, many more genes were downregulated in the array analysis than upregulated, again highlighting the importance of the increases in our mesenchymal markers. It is likely that all of these factors work in concert to promote a migratory and EMT-like phenotype, and that small gains in expression of a number of EMT genes can contribute to a greater overall effect.

The relationship between cyclin D1 expression and patient outcome remains a controversial area, with studies reporting both positive and negative associations. *CCND1 *gene amplification has been related to poor disease outcome in ER-positive patients [51,52], but others correlate cyclin D1 protein expression with both better [53,54] and worse [55] prognosis. It has been proposed that subgroup analysis with small numbers of patients [56] and splice variants of the gene have contributed to these contrasting results. In agreement with others [57], we found an association between high *CCND1 *expression and poor prognosis (Figure 3A). However, when examining *ID1 *high tumours, both the highest and lowest expression quartiles of *CCND1 *were correlated to reduced RFS/DFS but only in the ER-positive subgroup (Figure 4C). A similar trend was noted for *ID1*, where in all patients low expression of the gene was associated with a shortest RFS (Figure 3B), but in the *CCND1 *low ER-positive subgroup of tumours, a positive correlation was found (Figure 4B).

Whilst this may appear contrasting to our *in vitro *data, we reason that cyclin D1 low, ER-positive tumours best represent our cell line model. We chose two cell lines (MDA-MB-231 and ZR75-1) based on their high expression of cyclin D1 (regardless of oestrogen receptor status). We then reduced these high levels using siRNA and noted an increase in cell migration and EMT markers. As ER-negative tumours are consistently cyclin D1 low, these are less representative our *in vitro *experiments. ER-positive tumours however are typically cyclin D1 high, thus by choosing tumours that are cyclin D1 low in this subgroup, we are more correctly mimicking our *in vitro *setting, where expression of cyclin D1 may have been lost. This yields the interesting observation that ER-positive tumours with low cyclin D1 appear to behave similarly to ER-negative tumours with regards to their relationship to EMT markers and the claudin-low subtype. Thus, should ER-positive tumours that have lost expression of cyclin D1 be considered more ER-negative-like? Whilst the answer to this question is far beyond the scope of this study, what is clear is that the effect we are observing is centred on loss of cyclin D1 and not on the oestrogen receptor status of our testing material.

Interestingly, the *CCND1*^low^/*ID1*^high ^and *CCND1*^high^/*ID1*^high ^tumours both displayed increased expression of EMT-related genes (Figure 4A, yellow and green bars respectively). This suggests that in the context of these subgroups, *ID1 *is vital for increased EMT gene expression and when *CCND1 *is low it enhances the EMT phenotype.

We did not observe any meaningful impact of EMT genes in individual Kaplin-meier analysis on patient survival in our dataset. There has been an explosion of EMT related data in recent years in the breast cancer field. Central to many of these publications has been the ability of EMT to putatively enhance stem cell-related features and promote the metastatic process [58,59]. Of particular note, the idea of cells that have undergone EMT residing at the leading edge of an invasive tumour and promoting metastasis at the tumour- stroma interface has garnered much attention [60]. This hypothesis may be one explanation as to why EMT markers such as *SNAI1*, *SNAI2*, *TWIST1 *and *VIM *do not show any prognostic significance in our model- if the cells that have undergone EMT reside at the leading edge of the tumour, strong expression of their genes could easily be lost amongst the entirety of the tumour body. In these circumstances, any strong links to prognosis would also be diluted.

A second, more straightforward explanation as to why we have not observed prognostic significance of EMT- related genes centers upon a keystone principal. Upregulation of one EMT gene, e.g. *SNAI1*, is not enough to induce a transition to mesenchymal phenotype. This is supported by the board range of expression values of EMT genes across all breast cancer tumours and subtypes in our study (Figure 5D). Induction of EMT requires a reduction in *CDH1 *expression and upregulation of the potent *SNAI1, SNAI2 *and *TWIST1 *genes (amongst others). In order to examine the effect of EMT in our cohort, we would have to combine all tumours with these gene properties- giving us a 'claudin-low' subgroup. Unfortunately, we have too few cases in our claudin-low dataset to give any relevant prognostic information. In order to explore this further a cohort consisting of a large representation of claudin-low tumours, preferably with micro-dissection of the tumour-stroma interface would be required.

Much like *CCND1*, some controversy surrounds expression patterns of *ID1*, and despite numerous links to invasion and migration in breast cancer [43,44] some groups report an absence of the protein in the normal mammary gland [61]. Perk *et al. *assessed Id1 protein expression in mammary carcinomas [62] and found nuclear expression of Id1 in a rare subtype of breast cancer, metaplastic mammary tumours. Metaplastic cancers have a unique genetic profile that is notably, most closely related to the claudin-low subtype of breast cancer [41,63,64] and are very poorly differentiated. Given the poor outcome associated with metaplastic cancer, it may indicate why high *ID1 *expression in *CCND1 *low tumours gave the shortest RFS.

Adding further weight to our analysis, we found the greatest proportion of *CCND1*^low^/*ID1*^high ^cell lines and tumours in the claudin-low subgroup, which have a poor prognosis [65], associations with EMT and chemotherapy resistance [66] and has stem-cell tumour initiating features [42]. A number of these properties are reflected in both the cell lines and patient material used within this study, potentially indicating a central role for cyclin D1 and Id1 in this subgroup.

## Conclusions

The increase in MDA-MB-231 migration we have observed following cyclin D1 silencing is dependent on an upregulation of Id1 and induction of a more mesenchymal phenotype. Patients with *CCND1*^low^/*ID1*^high ^tumours have a shorter RFS and we have shown a link between *CCND1*^low^/*ID1*^high ^tumours and the claudin-low subgroup of breast cancer.

## Competing interests

The authors declare that they have no competing interests.

## Authors' contributions

NT performed the experimental work and the statistical analyses as well as drafting the manuscript. KL helped with the experimental work, and statistical analysis as well as drafting the manuscript. SL performed migration assays and western blots. AHS performed the statistical analyses and the bioinformatics related experimental work, as well as drafting the manuscript. GL was the principal investigator of the study and participated in the study design and interpretation of the data and helped to draft the manuscript. All authors read and approved the final manuscript.

## Pre-publication history

The pre-publication history for this paper can be accessed here:

http://www.biomedcentral.com/1471-2407/11/417/prepub

## Supplementary Material

Additional file 1 **qPCR primers**. Sequences of primers used in this study. Click here for file

Additional file 2 **TGF-β gene expression in breast cancer cell lines**. The dataset from Neve *et al. *was employed to examine TGF-β gene expression in breast cancer cell lines. The bar at the bottom of the figure represents the subtype of each cell line. Blue = luminal, Orange = Basal A, Red = Basal B. Cell lines of interest are highlighted with a black rectangle, and are ZR75-1 and MDA-MB-231 cell. Click here for file

Additional file 3 **Cyclin D1 silencing does not increase MDA-MB-231 cell migration in the absence of Slug**. Actively cycling MDA-MB-231 cells were monitored 20 h post-transfection with the indicated siRNA (cyclin D1/slug) for changes in cell migration and gene expression. Error bars represent standard deviation. **(A) **Cell migration as measured by Boyden chamber assay **(B) **qPCR analysis of slug expression. ***P ≥ 0.001, **P ≥ 0.01, *P ≥ 0.05 vs. control, two-tailed student's t-test. Click here for file

Additional file 4 **Correlation of *CDH1*, *VIM *and *TWIST1 *expression to recurrence free survival**. Expression of our genes of interest in relation to recurrence free survival was examined in a breast cancer meta-analysis. **(A) ***CDH1 *quartiles **(B) ***VIM *quartiles **(C) ***TWIST1 *quartiles. P-value is based on log-rank test. Click here for file
