# Supplementary material for: Cyclin D1, Id1 and EMT in breast cancer
Source: BMC Cancer. 2011 Sep 28;11:417. doi: 10.1186/1471-2407-11-417 (PMC3192789; doi:10.1186/1471-2407-11-417)
Supplement: Additional file 1 — qPCR primers. Sequences of primers used in this study. [file 1471-2407-11-417-S1.DOC]

| **Name** | **Direction (5’ to 3’)** | **Sequence** |
| --- | --- | --- |
| ***CCND1*** | Forward | CCTGTCCTACTACCGCCTCA |
|  | Reverse | CAGTCCGGGTCACACTTGA |
| ***ID1*** | Forward | CCAGAACCGCAAGGTGAG |
|  | Reverse | GGTCCCTGATGTAGTCGATGA |
| ***SNAI2*** | Forward | TTTTGCCAATTAACAGTATGTGC |
|  | Reverse | GCCAGGAATGTTCAAAGCTAA |
| ***B2M*** | Forward | TTCTGGCCTGGAGGCTATC |
|  | Reverse | TCAGGAAATTTGACTTTCCATTC |
| ***TUBB*** | Forward | TTAACCATGAGGGAAATCGTG |
|  | Reverse | CTGATCACCTCCCAGAACTTG |
| ***PGK*** | Forward | CTGTGGCTTCTGGCATACCT |
|  | Reverse | CTTGCTGCTTTCAGGACCA |
| ***Mrg1 Promoter*** | Forward | GATCCTTCAGTGAGCCAAGG |
|  | Reverse | ATGTGTTGCTGACCATCCAA |

**Supplemental Table 1.** qPCR and ChIP Primers
