# Supplementary figures and images for: Cyclin D1, Id1 and EMT in breast cancer
Source: BMC Cancer. 2011 Sep 28;11:417. doi: 10.1186/1471-2407-11-417 (PMC3192789; doi:10.1186/1471-2407-11-417)

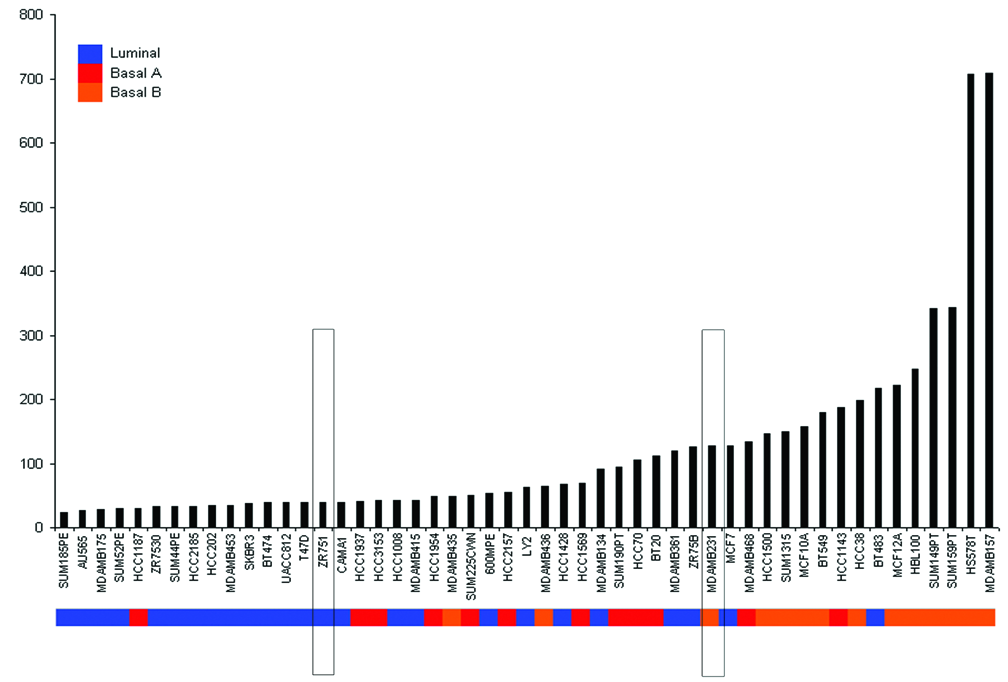

Supplement: Additional file 2 — TGF-β gene expression in breast cancer cell lines. The dataset from Neve et al. was employed to examine TGF-β gene expression in breast cancer cell lines. The bar at the bottom of the figure represents the subtype of each cell line. Blue = luminal, Orange = Basal A, Red = Basal B. Cell lines of interest are highlighted with a black rectangle, and are ZR75-1 and MDA-MB-231 cell. [file 1471-2407-11-417-S2.TIFF]

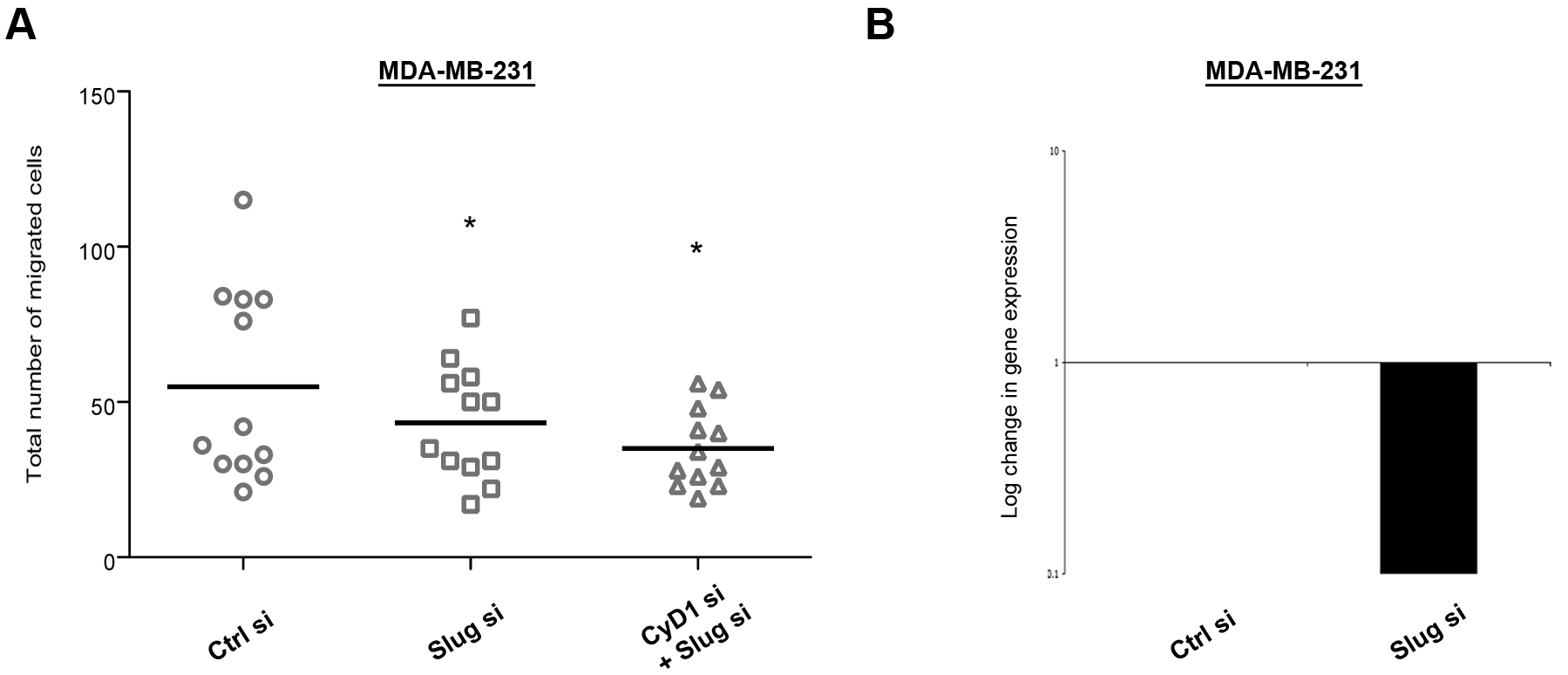

Supplement: Additional file 3 — Cyclin D1 silencing does not increase MDA-MB-231 cell migration in the absence of Slug. Actively cycling MDA-MB-231 cells were monitored 20 h post-transfection with the indicated siRNA (cyclin D1/slug) for changes in cell migration and gene expression. Error bars represent standard deviation. (A) Cell migration as measured by Boyden chamber assay (B) qPCR analysis of slug expression. ***P ≥ 0.001, **P ≥ 0.01, *P ≥ 0.05 vs. control, two-tailed student's t-test. [file 1471-2407-11-417-S3.TIFF]

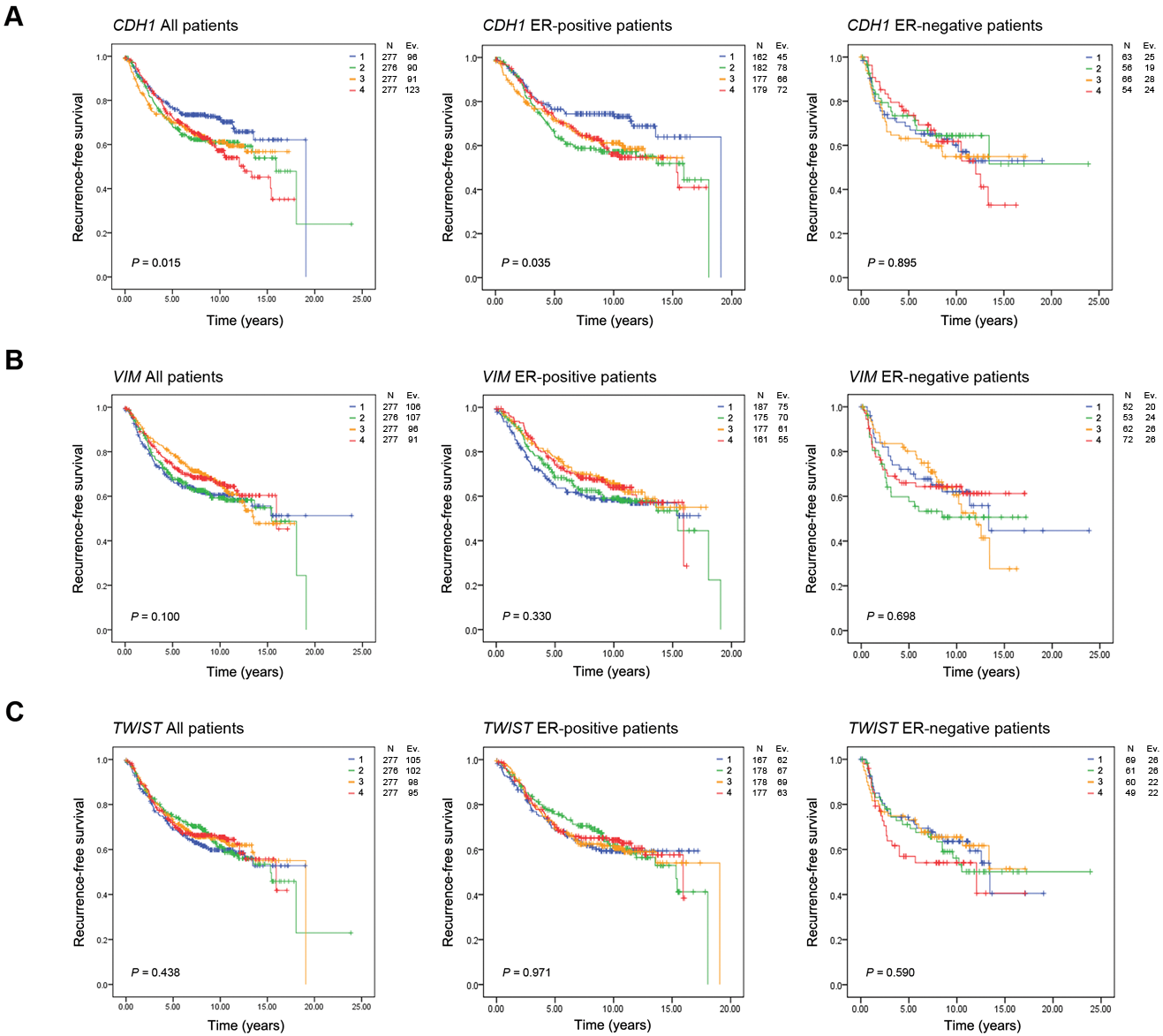

Supplement: Additional file 4 — Correlation of CDH1, VIM and TWIST1 expression to recurrence free survival. Expression of our genes of interest in relation to recurrence free survival was examined in a breast cancer meta-analysis. (A) CDH1 quartiles (B) VIM quartiles (C) TWIST1 quartiles. P-value is based on log-rank test. [file 1471-2407-11-417-S4.TIFF]
